# Singing the Individual: Name Tunes in Oyda and Yopno

**DOI:** 10.3389/fpsyg.2021.667599

**Published:** 2021-08-11

**Authors:** Azeb Amha, James Slotta, Hannah S. Sarvasy

**Affiliations:** ^1^African Studies Centre Leiden, Leiden University, Leiden, Netherlands; ^2^Department of Anthropology, University of Texas at Austin, Austin, TX, United States; ^3^The MARCS Institute for Brain, Behaviour and Development, Western Sydney University, Penrith, NSW, Australia

**Keywords:** Oyda, Yopno, surrogate speech, name tune, *konggap*, *moyzé*, music, whistled language

## Abstract

Music beats spoken language in identifying individuals uniquely in two disparate communities. In addition to their given names, which conform to the conventions of their languages, speakers of the Oyda (Omotic; SW Ethiopia) and Yopno (Finisterre-Huon; NE Papua New Guinea) languages have “name tunes,” short 1–4 s melodies that can be sung or whistled to hail or to identify for other purposes. Linguistic given names, for both communities, are often non-unique: people may be named after ancestors or contemporaries, or bear given names common to multiple individuals. But for both communities, name tunes are generally non-compositional and unique to individuals. This means that each new generation is likely to bring thousands of new name tunes into existence. In both communities, name tunes are produced in a range of contexts, from quotidian summoning and mid-range communication, to ceremonial occasions. In their use of melodies to directly represent individual people, the Oyda and Yopno name tune systems differ from surrogate speech systems elsewhere that either: (a) mimic linguistic forms, or (b) use music to represent a relatively small set of messages. Also, unlike some other musical surrogate speech traditions, the Oyda and Yopno name tune systems continue to be used productively, despite societal changes that have led to declining use in some domains.

## Introduction

Philosophers have long distinguished proper names from common nouns on the basis of their semantics; in contrast to common nouns, which denote classes of things, proper names are said to denote individuals (Searle, 1958; Kripke, 1980). In this study, we describe musical practices from different sides of the world that share this property with names. Among the Oyda of Ethiopia and the Yopno of Papua New Guinea, a distinct musical tune is closely associated with each individual. In fact, these tunes are more uniquely associated with individuals than proper names are. In these communities, multiple individuals may share the same proper name, by virtue of being named after them, or by the name's popularity. What we term “name tunes,” in contrast, are unique to individuals, at least normatively.

Speech surrogate systems are often divided into two main types: abridgment and ideographic (Stern, 1957). In abridgment systems, musical features correlate with features of the affiliated language: pitch with tone or vowel quality, or duration with vowel length. This is typical of “whistled languages,” such as those corresponding to Spanish, Turkish, and Highland Mazateco (Cowan, 1948; Classe, 1957; Meyer, 2021), but abridgment systems in which musical instruments serve as proxies for speech are also attested (Niles, 2010). In ideographic systems, musical phrases are conventionally associated with words or expressions, bearing meaning without a phonic connection to the language. These are typified by some of the slit-gong drum messaging traditions of northern New Guinea (Burridge, 1959; Niles, 2010), and the “talking drums” of West Africa (Neeley, 1999; Winter, 2014).

The Oyda and Yopno name tune systems differ from both abridgment and ideographic systems in that the name tunes directly denote an individual, without recourse to language. That is, Oyda and Yopno name tunes reference individual human community members themselves; they are not “abridgments” or “ideographs” of people's given names. While there are minor stylistic differences between Oyda and Yopno name tunes, the two traditions are remarkably similar, and both are absent from most surrounding communities. This paper offers a first comparative analysis of the usage contexts and formal characteristics of Oyda and Yopno name tunes.

## Comparing Oyda and Yopno Name Tune Traditions

### Oyda Name Tunes: *moyzé*

The Oyda language of southern Ethiopia (about 45,000 speakers) belongs to the Ometo branch of Omotic. The Oyda region is dominated by mountains and valleys which are partly shaped by the Great Rift Valley system.

Most Oyda people have both a proper name and a *moyzé* “name tune.”^1^ Both men and women, as well as children from age five or six onward, use *moyzé*. While a proper name can simultaneously be used to designate different people, two or more living people cannot share the same *moyzé*. As such, *moyzé* are a more reliable personal identifier than a proper name.

*Moyzé* are typically whistled by blowing air between two fingers of a hand held against the mouth while the airstream is modified by the movement of fingers of the other hand (Amha in preparation). Some people blow air at a joint between their index-finger and middle finger, some between the middle finger and their ring-finger, still others between their ring-finger and little finger. This appears to have impact on the audibility of the whistle: air blown into the juncture between the index finger and the ring finger seems to have higher pitch, and is said by the Oyda to “reach farther,” than that between the index finger and ring finger. Another difference among people involves the hand someone habitually uses to blow air into. Some prefer to use the left hand for this while using the right hand for modulating air; others prefer the reverse. People claim that they can tell who is whistling their *moyzé* name based solely on the sound. According to one consultant: “The whistling of different people differs as their fingerprint would differ.”

An individual's *moyzé* is known and used by people close to him/her and is also introduced to new acquaintances. Such “teaching of *moyzé*” is done by singing the *moyzé* using consonant-vowel (CV) combinations, such as *léeteléetetóom*, for someone whose proper name is S'as'ima. Such sung “vocables” are not used to address people but are crucial for transmission of *moyzé* forms. Sung vocables can include any of the five vowels of the language: /i/, /u/, /e/, /o/, and /a/. Both short and long forms are attested. Consonants tend to be /m/, /n/, /l/, /w/, /h/, and /t/, but a few *moyzé* containing /b/ and /g/ are recorded, e.g., *tóógirgidáal*í*iim*.

*Moyzé* are most often used for communication at a distance, but also serve to invoke the memory of a deceased person. *Moyzé* are often used to summon or get the attention of family members at work in different places, to alert the addressee of some danger or emergency, and to greet someone when passing by his/her house. Until hunting was outlawed in the 1970s, *moyzé* were also widely used to assemble people for a hunting party. Among Oyda who still practice the traditional religion, *moyzé* play an important role in announcing death to family members in different villages and during funerals, where the *moyzé* name of the deceased is repeatedly called using a side-blown, trumpet-like instrument, also called *moyzé*, that is made from cattle horn. Indeed, it is possible that the term originally derived from *moys-* ‘to see off, accompany someone for part of the journey,' and that the original function of Oyda name tunes was funerary. With the expansion of Protestant and Pentecostal faiths among the Oyda, the use of *moyzé* in funeral contexts is eroding, but its use in daily communication has been maintained. In some cases, young people may be innovating new arenas in which to use *moyzé*; one young man uses his deceased father's *moyzé* as a mobile ring tone.

*Moyzé* may be given (e.g., by a parent to a child, a husband to his wife), may be self-created, or inherited. Adults can choose to keep an inherited *moyzé* of a deceased family member alongside their own *moyzé*, thereby having more than one *moyzé* for some time. Either their own *moyzé* or the inherited one can then be passed on to one of their own descendants.

### Yopno Name Tunes: *konggap*

The Yopno language (Reed, 2000; Slotta, 2014) is spoken by some 8,000 people living in the Yopno valley in the northeast of Papua New Guinea. Yopno is a Papuan language of the Finisterre branch of the Finisterre-Huon language family. Yopno is most closely related to the Nankina and Domung languages to its north and west. Speakers of these three languages (total population: 15,000) all use name tunes, known in Yopno as *konggap* (Nankina: *kunggwap*). *Konggap* have been discussed by Niles (1992), Slotta (2012), and Ammann et al. (2013).

*Konggap* are most often sung using the vowels /a/, /o/, and /e/. Sung in this way, *konggap* can be heard at great distances across the deep gorges and along the steep mountain slopes found throughout the region. This is also the form in which *konggap* are performed at funerals and in ceremonial dances. But on a daily basis in the confines of homes, villages, and gardens, *konggap* may also be whistled, hummed or sung in the middle of spoken conversation.

Virtually everyone in the region has one of these melodies uniquely associated with him or her. The *konggap* are sung, whistled, and hummed throughout the day as people summon others, alert them to their presence, or even think about them. The Yopno language is non-tonal and *konggap* bear no phonic relation to people's proper names. Rather, each is a melodic and rhythmic sequence that identifies its bearer uniquely in a way that proper names do not, a point Yopno people routinely mentioned to the second author when discussing *konggap*. Traditional Yopno proper names are typically shared with living namesakes or ancestral forebears. Today, these names are often supplemented with names from English or Kâte, the former lingua franca of the Lutheran church in the region. But these too are drawn from a limited stock, so it is common for people to share a name with numerous others. In contrast, *konggap* are described as ideally unique to their bearer.

When still a baby, a child receives its first *konggap*, often composed by the mother while tending to the child. That *konggap* is later replaced by a new melody composed by a friend, relative, or often by the bearer him- or herself. The *konggap* of some is given by ancestors in dreams. Over their lives, people may cycle through multiple *konggap*, and even have more than one in use at the same time.

On a day-to-day basis, *konggap* are perhaps most often used to hail and summon, but, as with the funerary *moyzé*, they can also bespeak emotional associations with the people whose *konggap* are sung. Passing by a relative's house on the way to the forest, a man will sing his relative's *konggap* to alert him to his own passage. Seeing a friend at a distance along the steep mountain slopes in the region, a person will sing the friend's *konggap* as a greeting or summons. When one's thoughts turn to a person who has died or who one has not seen for a while, one expresses sorrow by singing or whistling the relative's *konggap*. Joy at the arrival of a friend is expressed with *konggap*. Shown a photo of the local preschool class by the second author, a group of children in Nian village burst into a mass of song—each singing out the *konggap* of a friend they recognized in the photo.

As with *moyzé, konggap* play a role in announcing death. Here, however, care must be taken, because singing a deceased person's *konggap* can also be taken as a sign that the singer is responsible for their death. At a funeral, where people gather around the body of the deceased for one or more days, women collectively sing the *konggap* of the deceased and of the relatives and friends of the deceased, sometimes cycling through dozens of *konggap*. The name tunes often elicit tears in the listening mourners.

*Konggap* are also sung in men's ceremonial dances (Niles, 1992; Ammann et al., 2013) to mark other moments of social importance—a new marriage, the conception of a first child, or an official event. A group of men sing their own individual *konggap* simultaneously, synchronizing the beginnings and endings of their very different *konggap* with a common beat. This is one of the few times a person sings his own *konggap*, which otherwise rings of great pretension. Indeed, an individual performer's *konggap* is said to potentially take on magical potency to seduce women in such performances.

Table 1 compares key features of the *konggap* with those of the *moyzé*.

**Table 1:** Comparative summary, *moyzé*, and *konggap* traditions.

	***moyzé***	***konggap***
Location	Oyda district, Gofa zone of the Southern Nations, Nationalities, and Peoples Regional in Ethiopia	Nayudos LLG, Madang Province and the neighboring Yopno valley in Morobe province, Papua New Guinea
Environment	Parallel rolling hills, elevation between 1500 and 2600 m	Steep-sloped mountains and side valleys cut by streams flowing into the deep gorge shaped by the Yopno river, cloud rainforest and grassland, elevation between 1000 and 3000 m
Taboos on use of linguistic given names	Not strictly taboo. But often people are addressed using teknonyms, titles (“chief”) and relational terms (“sister,” “brother”) even when no kinship relation exists	Avoid the names of in-laws. Older relatives and members of the community are typically not addressed by name
Acquisition	Given by parents, self-created or inherited: used by age five or six	Mothers innovate name tunes to their infants during infancyLater, individuals can change their own name tunes
Whistling production method	Air blown into hand and modulated by fingers of other hand	Whistled without hands
Sung method	CV sequences; C = tends to be /m/, /n/, /l/, /w/, /h/, /t/, but /b/, /g/ are also used. All five vowels of the language, /i/, /u/, /e/, /o/ and /a/ as well as their long counterparts are used	V sequences only: primarily /e/, /a/, /o/
Quotidian use	Hailing, summoning, alerting, recalling deceased	Hailing, summoning, alerting, recalling deceased
Special occasion use	In funerals, only through the use of a blown horn	Ceremonial dance, funerals
Taboos	Two living people cannot have similar-sounding *moyzé* names	Aside from ceremonial dances, people are reluctant to sing their own *konggap*
More than one per person?	Yes, possible: some may inherit a *moyzé* while they have their own	Yes, possible: an individual may be associated with different *konggap* in different places

### Formal Qualities

**Figure 1:**
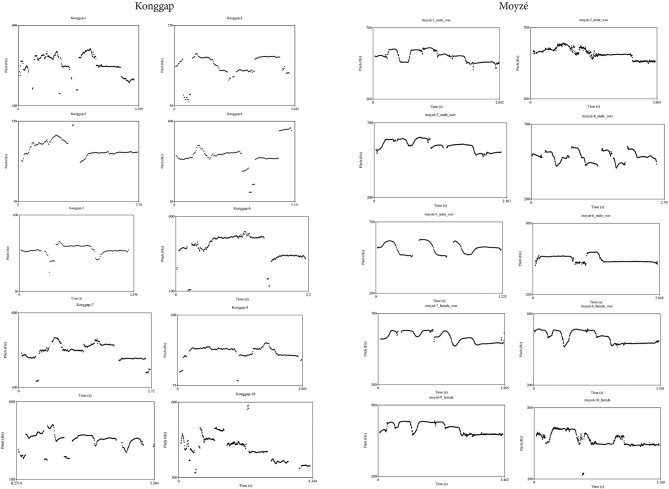
Pitch traces for 10 sung *konggap* and 10 whistled *moyzé* name tunes.

Figure 1 shows pitch traces for a sample of 10 sung *konggap* (left) and 10 whistled *moyzé* productions (right), analyzed using Praat (Boersma and Weenink, 2019). Time (in seconds) is charted on the x-axes, and pitch range (Hz.) is charted on the y-axes. The *konggap* were recorded using a Sony MZ-R55 minidisc recorder at the funeral for a deceased woman. In the recordings, a group of women sing the *konggap* of relatives of the deceased, using the vowels /a/, /e/, and /o/. The *moyzé* were recorded using a Linear PCM LS-11 audio recorder at 44.1 kHz/16bit. Six were performed by men and four by women.

Figure 1 shows that the name tunes of both traditions often end with relatively lower pitch than the rest of the tune, and the final “note” is often sustained. These pitch contours are not directly reminiscent of the prosodic contours of any linguistic units in either language. Due to the unstaged group singing and noisy, uncontrolled recording context of the *konggap*, brief, spurious pitch trace fragments appear, primarily in the lower frequencies. These were not used for measuring pitch ranges, below.

**Figure 2:**
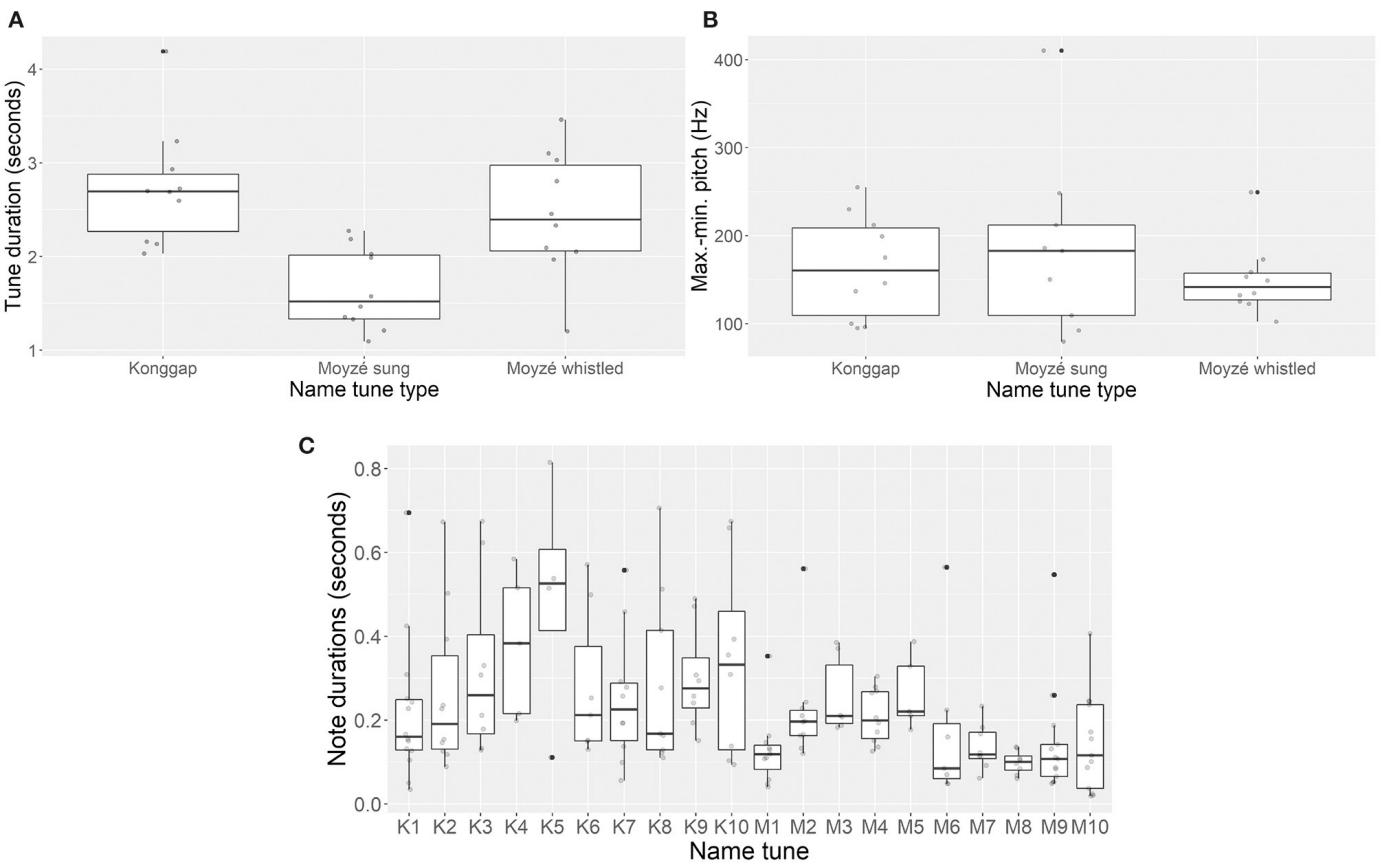
Tune duration, pitch range, and average note duration for sampled *konggap* and *moyzé* (*K* = *konggap, M* = *moyzé*).

Figure 2 visualizes tune duration, pitch range, and note durations of the productions in Figure 1, along with sung renditions of the same *moyzé*. Figure 2A compares durations of the *konggap* with those of both sung and whistled *moyzé*. The sung *konggap* and whistled *moyzé* have similar average durations; the sung *moyzé* are shorter than all *konggap*, and most whistled *moyzé*. Figure 2B gives the results of subtracting the least pitch value from the greatest pitch value in each tune, based on actual sung notes, not any spurious pitch readings by Praat that appear in Figure 1. The *konggap* and sung *moyzé* are very similar here, while whistled renditions of the *moyzé* feature smaller pitch ranges, overall. Note that the graph depicts the difference between minimum and maximum pitch in each name tune, not the actual pitch values.

Figure 2C aims to quantify the amount of rhythmic variation in each name tune. We segmented all “notes” in *konggap* and sung *moyzé* samples in Praat. A note was taken to be a sung vocoid or CV sequence with perceptually stable pitch; this was obtained by listening, along with visual inspection of the spectrogram. The number of notes per tune ranged from 4 to 14 (mean: 8.7; median: 8). Overall, there is more variation in note duration within individual *konggap* samples than within individual *moyzé* samples. Several *moyzé* are characterized by relatively even and short note durations (generally <0.4 s in duration), while all *konggap* include mixtures of short notes (lasting 0.2 s or less), and long notes (lasting 0.4 s or more).

In sum, *konggap* and *moyzé* are formally very similar. They last for similar amounts of time (1.09–4.19 s) and feature similar pitch ranges. *Moyzé* vary more in length than *konggap*, and *konggap* tend to have more internal rhythmic variation than the *moyzé*. Durations and pitch contours of both *konggap* and *moyzé* suggest that, if they should be compared with any linguistic units, this should be the utterance or clause (not the word, or proper name). Future research should compare the forms of *konggap* and *moyzé* to Yopno and Oyda musical genres: we expect that musical styles may reveal more than language about the origins of *konggap* and *moyzé* stylistic conventions.

## Discussion

This has been the first comparative analysis of two name tune traditions. We have seen that in the Oyda and Yopno name tune traditions, a short musical sequence references an individual community member. These name tunes are maximally specific and individual; more so than proper names within each language, which can be identical in both communities to either ancestral figures' names or to living people's names. It appears that each new generation induces the generation of thousands of new *moyzé* and *konggap* name tunes. These name tune systems thus exemplify the human capacity for musical creativity, as well as for recognition and reproduction of musical sequences.

The Oyda *moyzé* and Yopno *konggap* are similar to each other in length and pitch range per tune, but diverge slightly in internal rhythmic variation (greater in the *konggap*). In both traditions, name tunes play an important role in funerary proceedings, evoking the essence of the deceased individual in a medium that could be said to speak directly to the emotions. The Yopno name tune tradition differs from the Oyda tradition in that *konggap* are also used in men's group song/dance performances.

Musical evocation of individuals is found beyond these two systems. The *yoik* of the Sami are short songs or chants that can be not only linked to individual people, but also to activities, animals, emotions, and nature (Anderson, 1984; Krumhansl et al., 2000: p. 19; Hanssen, 2011). At least two of the northern New Guinea communities with slit-gong drum messaging traditions (the Tangu and Reite) are also reported to have used particular drum sequences for each individual person—and sometimes individual domestic animals—in a community (Burridge, 1959; Leach, 2002).

In contrast, the Mehek, a Sepik area New Guinea community (Hatfield, 2016) have a small, apparently fixed inventory of *isi* “name whistles,” which, crucially, are each associated with a given name, not with individual people. Individuals who share a given name also share an *isi*. Some *isi* also map onto multiple given names; only 94 *isi* are attested. The tunes themselves are generally shorter and less complex than either *konggap* or *moyzé*. Mehek further has a few longer name “songs” for some given names, which appear to be slightly longer and more complex than either *konggap* or *moyzé*.

Table 2 compares features of these other traditions with those of the *konggap* and *moyzé*.

**Table 2:** Comparison of Oyda and Yopno name tune systems with other surrogate speech systems.

		**Formal relationship with words in language**	**Message compositionality**	**Creative freedom**	**Number of distinct forms**
Abridgement systems	Whistled (Spanish, Turkish, Tepehuan, Hmong, Moba, and many others)	Phonic	Often extensive	Message creation	Somewhat less than the number of lexical items in the language
	Instrumental (Drum: Akan, Banda-Linda, Bora; Xylophone: Seenku; among others)	Phonic	Yes	Message creation	Less than the number of lexical items in the language
Ideograph systems	Instrumental (Senegalese drum language, New Guinea slit-gong signaling)	Lexical	Limited	Message creation	Significantly less than the number of lexical items in the language
	Mehek name whistles (*isi*), Papua New Guinea	Lexical	No	No	94 attested conventional whistled counterparts exist for certain given names (not individuals)
Name tunes	Oyda *moyzé*	None	No	Tune composition	Potentially tens of thousands: most individuals have a unique name tune
	Yopno *konggap*	None	No	Tune composition	Potentially tens of thousands: each individual ideally has a unique name tune

*Whistled Abridgement Systems (Classe, 1957; Rialland, 2005; Meyer, 2021 inter alia), Instrumental Abridgement Systems (Nketia, 1971; McPherson, 2018; Seifart et al., 2018 inter alia), Ideographic Systems (Burridge, 1959; Zemp and Kaufmann, 1969; Winter, 2014; Hatfield, 2016)*.

Mountainous environments may have played roles in the development of name tune systems among both the Oyda and the Yopno, as may be the case with surrogate speech systems—especially whistled ones—elsewhere (Meyer, 2021). Indeed, in both Oyda and Yopno, name tunes coexst with small inventories of whistled phrases (in Yopno, these may also be sounded on a conch shell horn) with meanings like “yes,” “come,” and “go”: these do correspond to the Oyda and Yopno languages through abridgment, just like other whistled languages around the world. Another factor in the development of these name tunes systems may be taboos on pronouncing certain proper names: in both communities, people avoid using some proper names, in either strict taboos on speaking in-laws' names (Yopno) or milder social preferences to address and reference using kin terms (Oyda). But such preferences are far from unique to these communities (Fleming, 2011). The fact remains that neighboring communities to the Oyda and Yopno, who live in similar terrains and have similar cultural practices, lack name tune traditions entirely.

The next populated area due east from the Yopno area is the Uruwa region. Although Uruwa people use no name tunes, just one of the 10 Uruwa villages uses birth-order terms (small sets of names that denote a person's sex and birth-order) to hail and refer to individuals, while the other villages do not (Sarvasy, 2013, 2017). Perhaps naming systems—musical or linguistic–lend themselves to clean breaks with neighboring groups, unlike other aspects of language and culture, which are more likely to evince clines.

## Data Availability Statement

Publicly available datasets were analyzed in this study. This data can be found at: https://dobes.mpi.nl/projects/oyda/language/.

## Ethics Statement

The studies involving human participants were reviewed and approved by Volkswagen Stiftung for the DoBeS project on Oyda; University of Chicago Institutional Review Board, University of Chicago. Written informed consent for participation was not required for this study in accordance with the national legislation and the institutional requirements.

## Author Contributions

AA contributed to Oyda name tune data and analysis. JS contributed to Yopno name tune data and analysis. HSS contributed to formal analysis. All authors contributed to writing.

## Conflict of Interest

The authors declare that the research was conducted in the absence of any commercial or financial relationships that could be construed as a potential conflict of interest.

## Publisher's Note

All claims expressed in this article are solely those of the authors and do not necessarily represent those of their affiliated organizations, or those of the publisher, the editors and the reviewers. Any product that may be evaluated in this article, or claim that may be made by its manufacturer, is not guaranteed or endorsed by the publisher.

## Footnotes

^1^ https://hdl.handle.net/1839/d63213ce-eab2-41cd-ae9a-ff3f4a8b1f40
